# Dispersive solid phase extraction using a hydrophilic molecularly imprinted polymer for the selective extraction of patulin in apple juice samples

**DOI:** 10.1007/s00604-023-06056-8

**Published:** 2023-11-25

**Authors:** Chiara Cavaliere, Andrea Cerrato, Aldo Laganà, Carmela Maria Montone, Susy Piovesana, Enrico Taglioni, Anna Laura Capriotti

**Affiliations:** Dipartimento di Chimica, Università di Roma La Sapienza, Piazzale Aldo Moro 5, 00185 Rome, Italy

**Keywords:** Dopamine-formaldehyde–melamine MIP, Uric acid dummy template, LC-DAD, UHPLC-MS/MS, Mycotoxins, Fruit juice analysis

## Abstract

A molecularly imprinted polymer with a specific selectivity for patulin was successfully synthesized. The molecularly imprinted material was prepared using the two functional monomers dopamine and melamine and formaldehyde as the cross-linker. The resulting material possessed a large number of hydrophilic groups, such as hydroxyls, imino groups, and ether linkages. For the first time, uric acid was used as a dummy template for its structural similarity to patulin. Comprehensive characterization and detailed studies of the adsorption process were carried out via adsorption isotherms, while the rate-limiting steps were investigated using adsorption kinetics. Separation, determination, and quantification of patulin were achieved by ultra-high performance liquid chromatography coupled with both photodiode array detection and tandem mass spectrometry. The latter was applied to patulin confirmation in the analysis of real samples. The methodology was validated in 20 apple juice samples. The results showed that the developed hydrophilic molecularly imprinted polymer had high selectivity and specific adsorption towards patulin, with mean recoveries ranging between 85 and 90% and a relative standard deviation lower than 15%. The developed molecularly imprinted polymer exhibited good linearity in the range 1–100 ng mL^−1^ with coefficient of determination (*R*^2^) > 0.99. The limit of detection was 0.5 ng mL^−1^, and the limit of quantification was 1 ng g^−1^. The developed method showed a good purification capacity for apple juices due to its hydrophilic nature and the polar interactions established with the target analyte.

**Graphical abstract:**

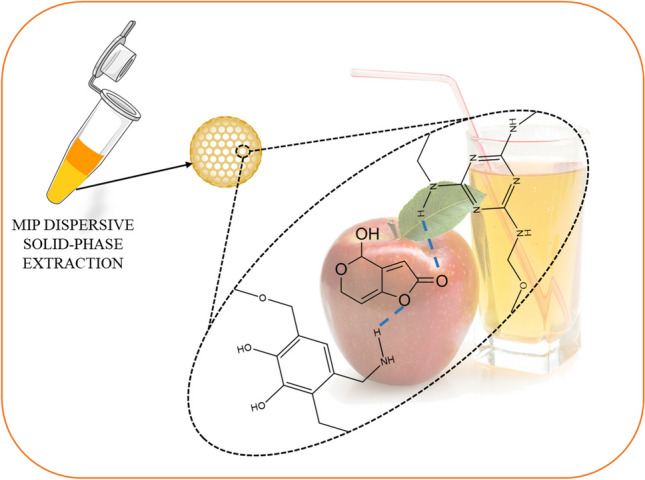

**Supplementary Information:**

The online version contains supplementary material available at 10.1007/s00604-023-06056-8.

## Introduction

Patulin (PAT) is an α,β-unsaturated γ-lactone mycotoxin generated as a secondary metabolite by at least 60 different species of fungi [1]. *Penicillium expansum* species is the most threatening one for people’s health and market security [2]. This species is often associated with a post-harvest disease affecting apples [3], although other fruits can be contaminated, including pears [4], citrus [5], and grapes [6]. Due to the presence of electrophilic hemiacetal and lactone rings [7], PAT is responsible for mutagenic effects and potential carcinogenicity [7–9], via covalent bonding with thiol groups of proteins or glutathione by Michael addition [10]. As such, the European Commission, through its Commission Regulation No 1881/2006, established maximum levels of PAT in foodstuffs. The maximum level is 10 μg kg^−1^ in apple juice and solid apple products, including apple compote and apple puree, for infants and young children; the limit is set at 50 μg kg^−1^ for fruit juice and 25 μg kg^−1^ for solid apple products (https://food.ec.europa.eu/safety/chemicalsafety/contaminants/catalogue/patulin_en.).

Due to the important health risks associated with exposure to PAT, the detection of even low levels of PAT in food, particularly in apple juices, is crucial [11] to ensure the safety and quality of apple juices and products [12]. There are individual challenges with the analysis of PAT. The physicochemical properties of PAT make it difficult to determine PAT because it is a highly polar and low-molecular weight molecule. Hydrophilic compounds such as PAT are difficult to retain on the column under reversed-phase conditions; therefore, a large water content in the mobile phase is usually required. For detection and identification of PAT in food, HPLC-UV is the technique of choice, due to the strong absorption of PAT. The main limitation of this approach is that specificity and sensitivity can be severely hampered due to the co-occurrence of 5-hydroxymethylfurfural (HMF), which produces interferences and affects the PAT quantification in HPLC-UV. PAT and 5-HMF have both strong UV absorbance and similar retention times, which often results in peak overlap. Many HPLC-UV studies took on the analytical challenge of improving patulin and 5-HMF peak separation. Nonetheless, the lack of sensitivity and specificity in HPLC-UV can be a serious disadvantage when low concentrations or complex matrixes are being analyzed, also due to interference of other compounds, such as phenolic compounds. Mass spectrometry could be a valuable alternative to improve the selectivity, but the low molecular weight of PAT makes it difficult to ionize and detect the molecule by this technique [12, 13]. This is particularly important when analyzing complex fruit matrices, for which the sensitivity required by regulations may not be met [14].

Many challenges associated with PAT detection can be dealt with by a dedicated sample preparation aimed at removing interfering compounds and reducing the matrix complexity. The efficiency of the extraction and clean-up steps improve the method’s sensitivity, precision, and specificity. In the last decade, several approaches were developed for the extraction and clean-up of PAT from complex samples, such as liquid-liquid extraction (LLE) [15–17], dispersion solid-phase extraction (d-SPE) [18], QuEChERS (Quick Easy Cheap Effective Rugged Safe) [19–21], and solid-phase extraction (SPE) [22, 23]. LLE, including vortex-assisted LLE, is the most common approach in HPLC-UV methods for PAT. Ethyl acetate extraction followed by sodium carbonate clean-up is the method suggested by the Association of Official Analytical Chemists. This approach has the drawback of causing PAT degradation during clean-up due to the high pH; therefore, alternative clean-up materials are needed [14]. New sorbents have been created as an alternative to commercially available SPE columns to improve the effectiveness of separating PAT from other components of the fruit matrix. The removal and pre-concentration of PAT from fruit products have been studied using a variety of polymers as solid-phase sorbents [14]. The major drawback of LLE and SPE procedures is that they are time-consuming, especially when large samples need to be analyzed [24]. QuEChERS utilizes a relatively high amount of extraction solvents and requires post-extraction cleaning, which can lead to increased preparation time, analysis costs, and potential risk of target compound loss [14].

Immunoaffinity columns could be a valuable tool for PAT purification due to their high selectivity and specificity [25]. Still, while different methods were developed for detecting many mycotoxins, including aflatoxins and ochratoxins [26, 27], no application was reported for PAT due to the high reactivity of PAT to thiol groups found in proteins, including antibodies. As an α,β-unsaturated γ-lactone, patulin can react with cysteine by Michael addition reaction and form the related adducts [28]. On the other hand, different molecularly imprinted polymers (MIPs) have been developed and widely used in the last decade for PAT clean-up in complex matrices before chromatographic analysis [29–38]. Commercial MIP materials for PAT are available [30]. In addition, dedicated materials were developed. Imprinting can be achieved directly using PAT as a template [39]; nevertheless, more often dummy templates are employed, to reduce template bleeding and improve safety in the production of materials. Oxindole was employed to imprint methacrylic acid-ethylene glycol dimethacrylate polymers via free radical polymerization in ethyl acetate [40]. Oxindole was also used to imprint silica beads in 83% methanol solution [31] and a silica shell on magnetic nanoparticles in 87% methanol solution [38]. To improve the selectivity, the combined use of two dummy templates was developed. Oxindole and 6-hydroxynicotinic acid were used to prepare methylacrylic acid-trimethylolpropane trimethacrylate polymers by radical polymerization in methanol [32]. The same combination of dummy templates was also employed to imprint a 4-vinyl pyridine-trimethylolpropane trimethacrylate shell on magnetic nanoparticles in methanol [41].

Most MIPs for PAT described in the literature were prepared in organic or organic-rich solvents, limiting the applications of MIPs in food, environmental, and clinical fields. In this work, the synthesis of a hydrophilic MIP in water/acetonitrile, 92:8 (*v*/*v*), was presented with dopamine and melamine as double functional monomers and formaldehyde as cross-linker, which introduce abundant hydroxyls, imino groups, and ether linkages into the material. Uric acid was used as a non-toxic dummy template of PAT; the resulting MIP showed good compatibility with water and excellent molecular recognition in extracting PAT in apple juices. The prepared polymer was characterized by scanning electron microscopy (SEM), Fourier transforms infrared (FTIR) spectroscopy, microanalysis, and Brunauer–Emmett–Teller (BET) analysis. In addition, static and dynamic adsorption was investigated to evaluate the adsorption performance. The MIP material was employed to develop and validate an analytical method for PAT analysis in apple juice samples with high-performance liquid chromatography (HPLC) and photodiode array detection (DAD). Finally, it was used to analyze *t* samples of apple juice. The selectivity to the common 5-HMF interfering compound was evaluated, and finally, PAT was confirmed in positive apple juice samples by UHPLC tandem mass spectrometry (MS/MS) analysis. The developed MIP was proved suitable for PAT monitoring, based on European legislation.

## Experimental section

### Chemicals and reagents

HPLC grade solvents, all solvents for sample preparation, acetic acid, dopamine, uric acid, formaldehyde solution (37%), and pure standards of all selected compounds, i.e., PAT (CAS 149-29-1), phenylalanine-d8 (CAS 17942-32-4), and caffeine-(trimethyl-^13^C_3_) (CAS 78072-66-9), were purchased from Merck Life Science (Darmstadt, Germany). Melamine was purchased by Fluka. The standard of PAT used as a certified reference material was 100 μg mL^−1^ in chloroform. A stock solution in methanol at a concentration of 2.5 μg mL^−1^ was prepared from this certified reference material. The concentration was verified using UV instrumentation (ɛ 14600, 276 nm). The stock solutions were diluted in water/methanol (90:10, *v/v*) to prepare appropriate working solutions. The working solutions were prepared weekly to prevent degradation, stored at −20 °C and brought to room temperature before use.

### Molecularly imprinted polymer and non-imprinted polymer preparation

Dopamine (30 mmol, 1.5 g) was mixed with formaldehyde (60 mmol, 1.5 mL) in 10 mL water at 40 °C by magnetic stirring for 1 h. In parallel, when the time was nearly over, melamine (10 mmol, 0.42 g) was added with formaldehyde (30 mmol, 0.766 mL) and stirred in 3.3 mL of water at 80 °C until the solution was clear. This latter mixture was then added to the former and stirred at 40 °C. For MIP preparation, the template uric acid was added (0.3 mmol, 50 mg), and the mixture was stirred for 30 min to dissolve the template and allow self-assembly. For comparison, the non-imprinted polymer (NIP) was also prepared by following the same procedure but without adding uric acid as a dummy template. Finally, 1.1 mL acetonitrile was added, and the mixture was left in static conditions at 40 °C for 1 day. When the reaction was over, the mixtures were left to cool at room temperature and washed with methanol/water, 4:1 (*v*/*v*) several times with and aid of vortex and ultrasound bath until supernatants were clear. Then, the resulting powder was dried at 40 °C overnight.

### Characterization of imprinted and non-imprinted polymers

The products were characterized by SEM (Tescan Vega) for morphological analysis, FTIR (IR spectrophotometer Nicolet iS50 coupled with a Nicolet Continuum FTIR microscope, Thermo Scientific) analysis for preparation assessment, and microanalysis (EA 1110 CHNS-O elemental analyzer, ThermoFinnigan). For SEM, sputtering coating with chrome was used to obtain images. For FTIR, spectra were acquired in the range 4000–650 cm^−1^, with a spatial resolution of 8 cm^−1^. Forty scans were acquired before elaboration by FT. Background spectra were subtracted for each measure. The surface area was determined by BET analysis (Micromeritics 3Flex 3500).

The thermodynamic and kinetic characterization of the prepared materials was studied by static and dynamic adsorption experiments, according to the description reported in Supplementary Information, section Adsorption experiments. The selectivity of PAT binding was also investigated against the main interferent compound 5-HMF, as previously described [38]. The detailed procedure is reported in the Supplementary Information, section Selectivity evaluation.

### Determination of PAT in apple juice samples

#### Sample collection

The apple juice samples (*n* = 20) were purchased from Italian markets, supermarkets, hypermarkets, or organic food stores. The samples were collected between November 2022 and January 2023. Juices of the same product and brand but from different batches were treated as separate samples. All the samples were stored in their original packaging at room temperature until the time of analysis. One aliquot was processed, and four were kept at −20 °C for analysis duplication.

#### Optimization of the dispersive solid phase extraction procedure

For method development, preliminary recovery experiments were performed to assess the most suited extraction conditions of PAT from spiked solvent mixtures. Experiments were performed on 100 mg of MIP, which was dispersed in 2 mL of a 7.5 ng mL^−1^ PAT sample in 0.1% acetic acid. The mixture was shaken at 25 °C for 30 min and then separated by centrifugation at 24 °C, 14,000 × g for 10 min. Elution was obtained by shaking with a proper solution for 10 min. Eluates were analyzed by HPLC-DAD as described in the final method.

##### Molecularly imprinted dispersive solid-phase extraction of apple juice samples

All samples were treated beforehand to eliminate the most abundant interferent compounds. Briefly, 15 mL of apple juice was acidified with 15 μL of acetic acid. Then, 25 mL of ethyl acetate was added. Samples were vortexed for 10 min. The mixture was then centrifuged at 8000 × g at room temperature for 10 min, the organic upper layer was collected, and the extraction was repeated twice. Subsequently, the three extracts were reunited and evaporated by a rotary evaporator. Residues were redissolved in 1 mL of a 0.1% acetic acid solution, 100 mg of MIP was incubated and vortexed at room temperature for 30 min; after the incubation time, the solution was eliminated, and the MIP was eluted with 1 mL of acetonitrile/acetic acid, 5:0.25 (*v/v*). The extracts were evaporated to a small volume (<50 μL) in a water bath at 37 °C under a gentle stream of nitrogen to remove most of the organic solvent while avoiding complete evaporation to preserve the integrity of the PAT. Then, samples were diluted in 200 μL water/acetonitrile, 95:5 (*v/v*) solution containing 30 μL of a 10 ng mL^−1^ solution of phenylalanine-d8 as internal standard. Finally, the concentration of PAT was determined by HPLC-DAD using the calibration curve.

##### High-performance liquid chromatography-photodiode array analysis

PAT was determined using a Shimadzu Nexera XR LC-20AD system, including a CBM-20A controller, two LC-20 AD pumps, and a DGU-20A3R online degasser. An SPD-M20A UV detector was used. Data acquisition was performed by the LabSolution version 5.53 software (Shimadzu, Kyoto, Japan). Chromatographic separation was carried out by reversed-phase chromatography on a column X-Bridge® Peptide BEH C18 (4.6 mm × 250 mm, 5 μm, Waters, Milford, Massachusetts, USA). Mobile phase A was water/acetonitrile, 90:10 (*v/v*), and mobile phase B was acetonitrile. Phase A was kept constant at 100% for 23 min, after which B was linearly increased from 0 to 70% within 5 min. Then, B was decreased to 0% and maintained at that level for the next 4 min to rinse the column. Aliquots of 100-μL samples were injected into the system manually. Separation was performed at a flow rate of 1 mL min^−1^. Detection was done by UV adsorption measurement at 276 nm. The retention time of PAT was ~14.45 min. All samples were analyzed in triplicate. Patulin was further confirmed by HPLC-MS analysis, as described in the section high-performance liquid chromatography-tandem mass spectrometry in the Supplementary Information.

##### Method validation

The HPLC-DAD method was validated following FDA guidelines, using an apple fruit juice pool. The parameters evaluated were as follows: recovery (RE), matrix effect (ME), precision, linear dynamic range, linearity, and the limit of detection and quantification (LOD and LOQ). Details on methods validation are reported in the Supplementary Information, section Method validation.

## Results and discussion

### Selection of template molecule

**Fig. 1:**
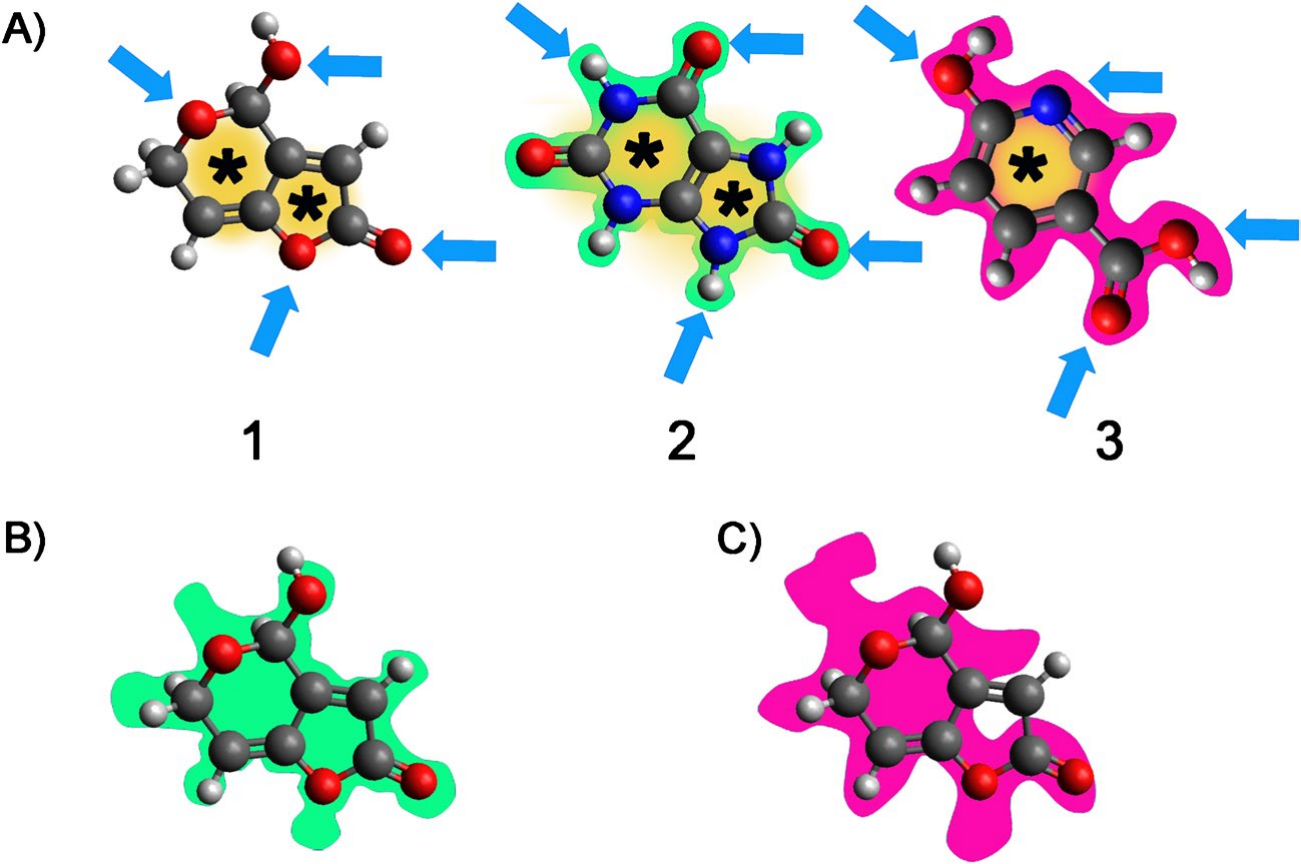
Schematic representation of the rationale for template choice. **A** Chemical structures of PAT (1) and the dummy templates uric acid (2) or 6-hydroxynicotinic acid. Molecular similar structures are highlighted by a yellow shade to indicate rigid structures and black stars indicate ring structures. Blue arrows indicate the potential sites for intermolecular interactions between the templates or PAT and the imprinted materials. Green and pink shapes are drawn to show the shape of the resulting imprinted sites for uric acid and 6-hydroxynicotinic acid, respectively. **B** Juxtaposition of the imprinted site for uric acid with PAT. **C** Juxtaposition of the imprinted site for 6-hydroxynicotinic acid with PAT. Molecular structures optimized geometries were drawn using Avogadro 1.2.0 (https://avogadro.cc/)

The proposed material represented an innovative d-SPE sorbent using a hydrophilic MIP to selectively extract PAT in apple juice samples. MIPs are typically prepared in organic or organic-rich solvents, thus exhibiting mediocre molecular recognition in aqueous mediums. To overcome such limits and expand their application in the food field, the synthesis of a hydrophilic MIP was based on a hydrophilic resin prepared in water according to previously described procedures with some modifications [42]. Specifically, the synthesis of hydrophilic MIP was achieved in a water/acetonitrile mixture with only 8% organic solvent. Dopamine and melamine were used as double-functional monomers and formaldehyde as a cross-linker, to introduce abundant hydroxyls, imino groups, and ether linkages into the material. The melamine-formaldehyde resin was also chosen to establish polar interactions with the analyte. Including dopamine in the reaction mixture would also improve interactions with additional sites. MIP was prepared with uric acid as a dummy template. The choice for uric acid was driven by the need to improve the selectivity by choosing a template with structural similarity to the target analyte, i.e., PAT. Although one is planar, the two molecules have similar shapes, with heteroatoms in similar positions (Fig. 1).

**Fig. 2:**
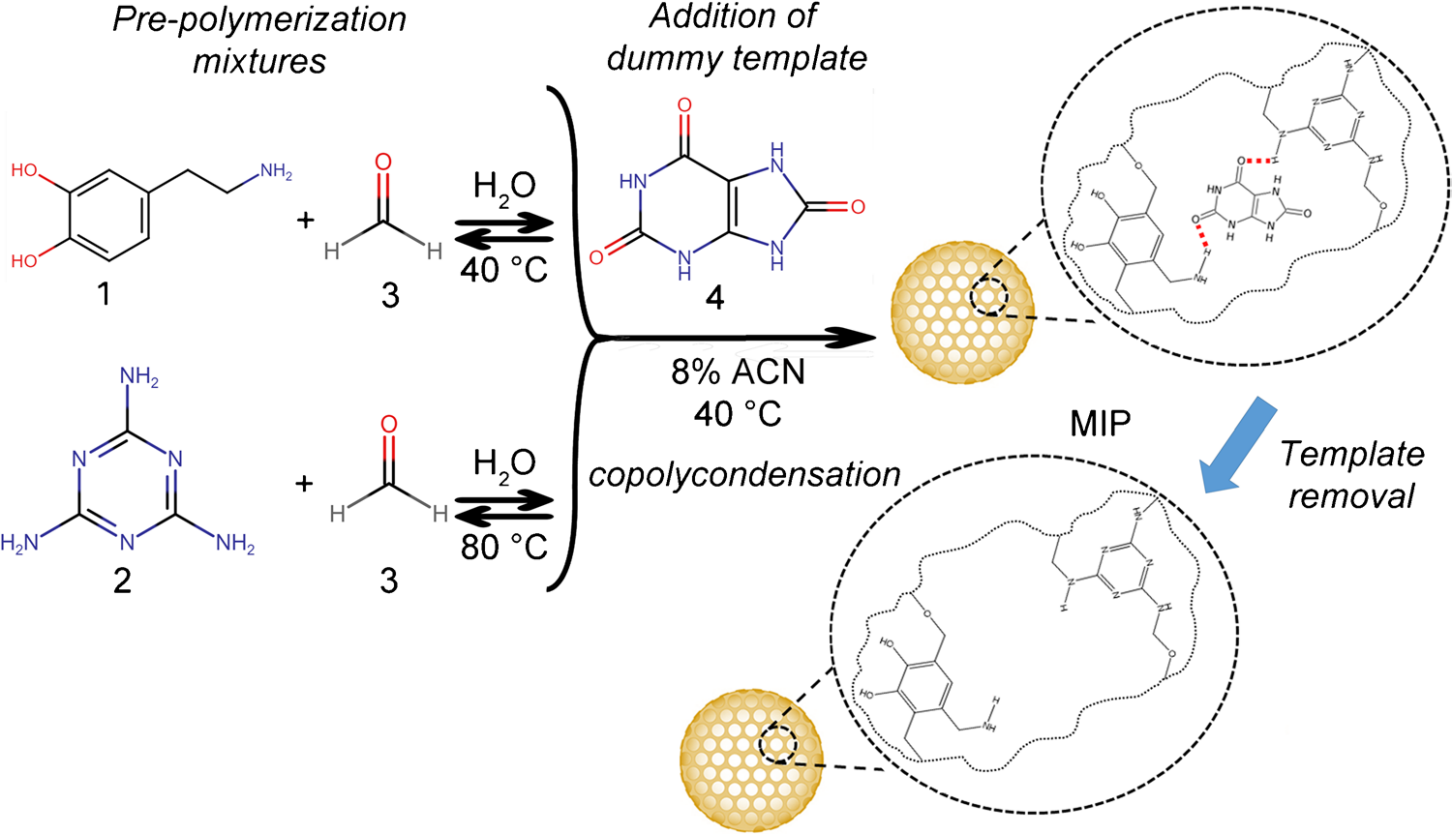
Schematic illustration of the synthesis steps of the MIP material. Dopamine (1) and melamine (2) are first reacted with formaldehyde (3) to prepare two pre-polymerization mixtures in water. The mixtures are then mixed and added with uric acid (4) dummy template and acetonitrile (ACN) to obtain a final 8% concentration of the porogen. The reaction is carried out for 24 h at 40 °C, to obtain the MIP material. After washing, the template is removed to obtain the final MIP product. A schematic representation of binding sites is also displayed. NIP material is prepared in the same way but without the addition of the dummy template

In the literature, reports describe MIP for PAT, and two main templates are used, i.e., 6-hydroxynicotinic acid, which is employed either alone or in a dual dummy template strategy with 2-oxindole [32, 43] and PAT [29]. We decided to look for a new template molecule and avoid using PAT, which could cause template bleeding and affect the analytical outcome. Moreover, as PAT is expensive and toxic, the use of a structural analog as a template molecule can reduce the toxicity and template leakage in the synthesis process of MIPs. Still, uric acid resembles PAT more than 6-hydroxynicotinic acid, which on the other hand, is a phenolic compound and can open to unwanted interactions with similar compounds abundant in fruits. Using uric acid as the template is expected to benefit the enrichment process’s selectivity by establishing polar and H-bond interactions with heteroatoms. Most of these interactions can be maintained when switching to PAT (Fig. 1B) while interactions are expected to be fewer in the case of 6-hydroxynicotinic acid (Fig. 1C). As such uric acid was considered as a good candidate to prepare a new MIP material with a different template molecule. Figure 2 shows a schematic representation of hydrophilic MIP’s synthesis and molecular recognition.

Melamine, a weak base, can accelerate the polymerization process by activation of phenol groups, so the formation of MIP can happen without adding base or acid, which ensures the success of molecular imprinting. Figure 2 clearly shows that dopamine can interact with the template via hydrogen bonding because of the hydroxyls of the monomer. Imino groups and amino groups in melamine can interact with the template via hydrogen bonding.

### Characterization of molecularly imprinted polymer

**Fig. 3:**
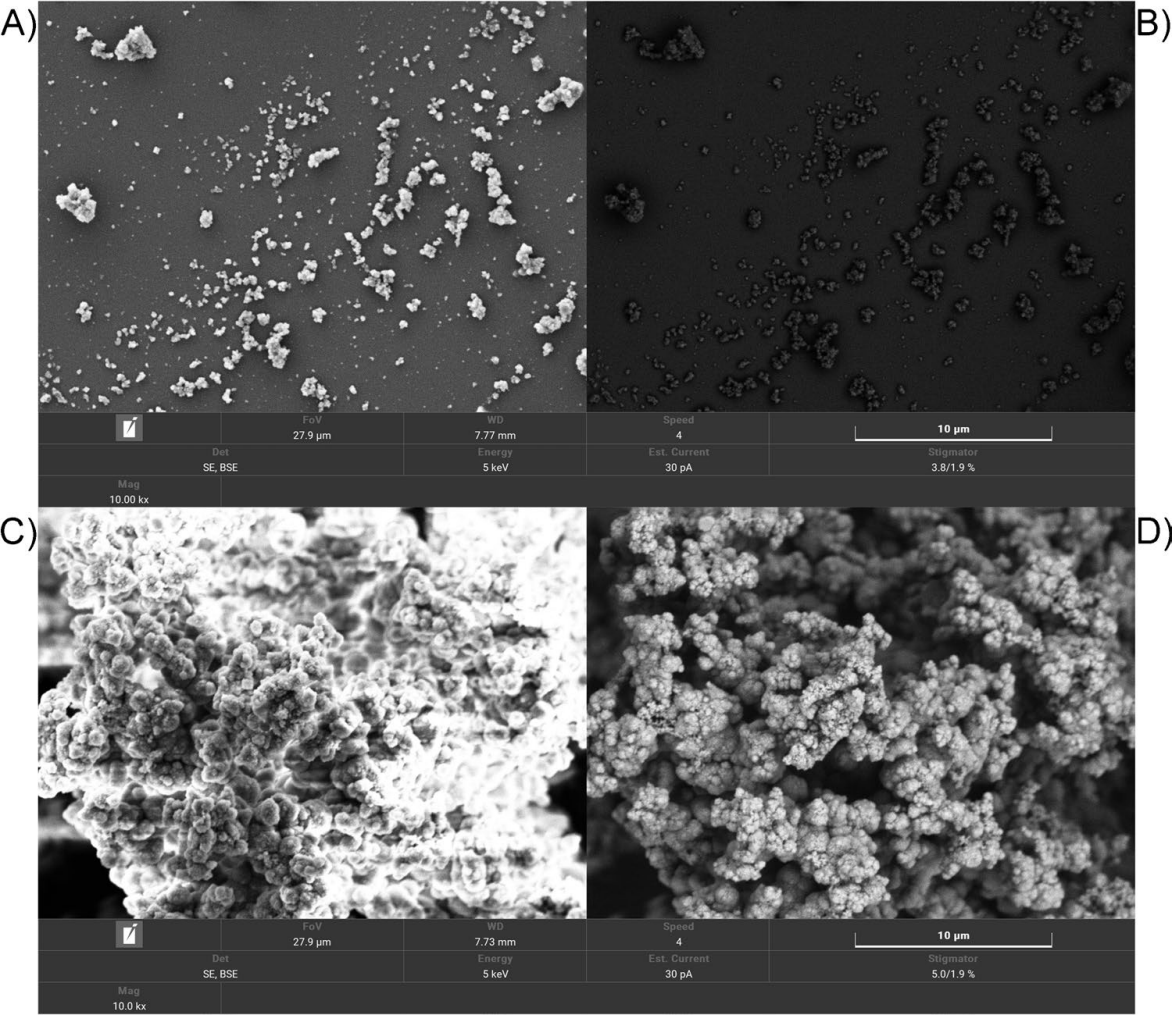
SEM images of the MIP (**A**, **B**) and NIP (**C**, **D**) materials at 10,000 magnification. **A**, **C** Pictures obtained by using a secondary electron detector. **B**, **D** The same images using a backscattered electron detector

The morphological analysis of MIP and NIP materials was done by SEM, which indicated that both materials are aggregates of smaller particles. The size of these aggregates reaches up to a few microns. The MIP looked more inhomogeneous than the corresponding NIP, which could be due to the influence of the template in the reaction mixture (Fig. 3, Fig S1).

**Fig. 4:**
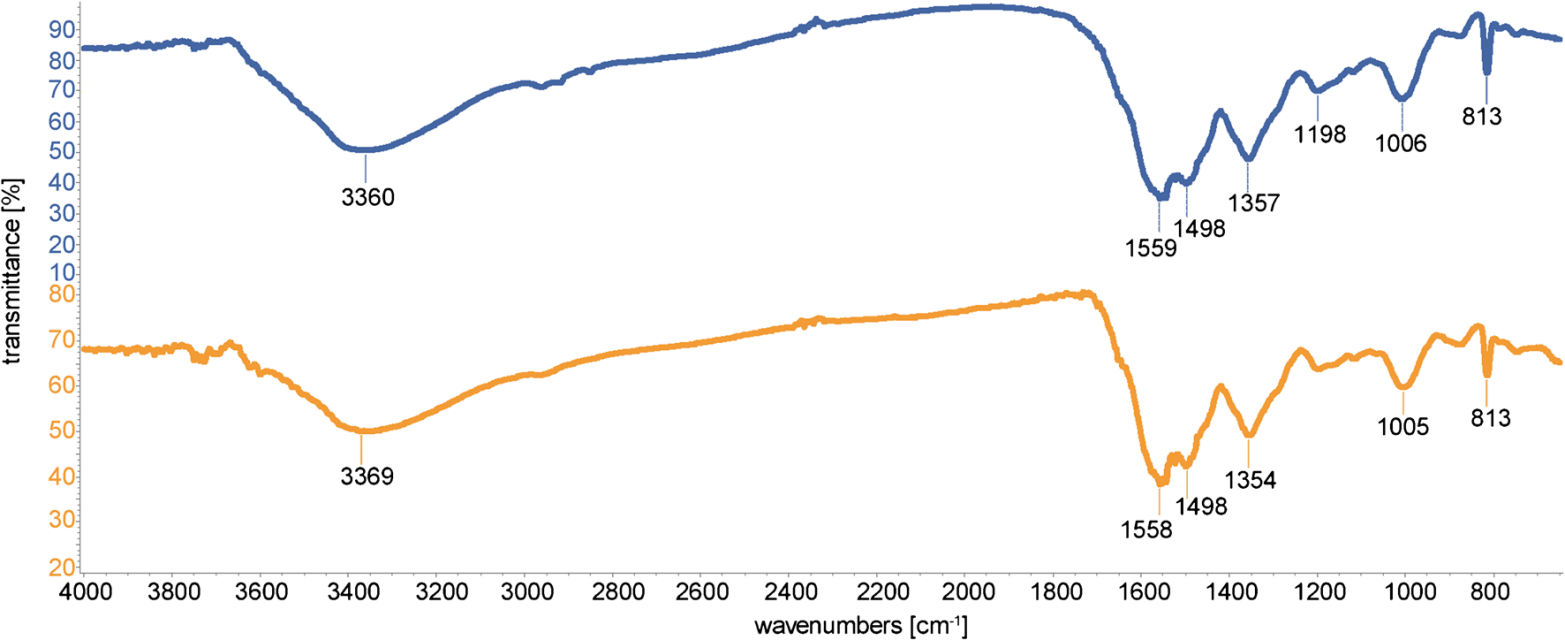
FTIR spectra of MIP (blue) and NIP (orange) materials. Images produced using Spectragryph (v1.2.16.1, http://www.effemm2.de/spectragryph/).

The FTIR spectra indicated that MIP and NIP were similar (Fig. 4).

The spectra agreed with previously reported similar materials [42]. The presence of two bands at ca. 1500 cm^−1^ and 1300 cm^−1^ was compatible with the aromatic stretching vibration and the ring-breathing mode of C–N in the triazine ring of melamine. Signals at ca. 1600 cm^−1^ were also compatible with C–C double bond stretching vibrations of aromatic rings. The strong and broadband at ca. 3400 cm^−1^ was compatible with the stretching vibrations of O–H and N–H; the band at ca. 1100 cm^−1^ was compatible with the out-of-plane bending of C–O–C. The close resemblance between MIP and NIP and the absence of strong bands at ca. 1700 cm^−1^ indicated that the template did not react with the monomers while preparing the MIP material. This was important because uric acids could also participate in the polymerization; however, using pre-polymerization with a controlled amount of formaldehyde allowed us to control the reaction and avoid the reaction of the template. The elemental analysis provided that the materials were made of 32% N, 38% C, and 5% H for both samples. Considering the possible products of the reactions, as described in [42] and [44], the elemental analysis was compatible with the reaction of one unit from the dopamine-formaldehyde adduct and two units from the melamine-formaldehyde adduct. Finally, the surface area was obtained by the BET method and was found to be 6.1 ± 0.5 m^2^ g^−1^ for the MIP material. Part of the surface area (i.e., 2.0 m^2^ g^−1^) was associated with micropores, whose total volume was 0.017 cm^3^ g^−1^ with a diameter of 0–800 Å and a maximum ≤ 17 Å. The BET analysis of the NIP provided similar values, with a surface area of 5.8 ± 0.5 m^2^ g^−1^. The micropores contributed 3.0 m^2^ g^−1^ to the surface area and had a total volume of 0.014 cm^3^ g^−1^, a diameter in the range of 0–800 Å, with a maximum ≤ 17 Å. The complete BET diagrams are displayed in the Supplementary Information (Fig S2).

## Adsorption studies

### Static adsorption and dynamic adsorption

Thermodynamic adsorption of MIP (Fig S3) and NIP (Fig S4) was investigated using different concentrations of PAT (5–350 ng mL^−1^). All experiments were performed under acidic conditions to prevent the degradation of PAT [20]. The adsorption amount of PAT on MIP material increased with increasing concentration and reached equilibrium at 250 ng mL^−1^ (Fig S5A). Two models were chosen to study the thermodynamics of adsorption, as described in the Supplementary Information, and the calculated *R*^2^ values were used to assess which model fitted the best (Table 1). The rebinding of PAT on the MIP material showed a better correlation with the Langmuir model, with an *R*^2^ of 0.98 (Fig S3A) vs. only 0.53 (Fig S3B) of the Freundlich model. These results demonstrated that the polymer recognition sites were evenly distributed as a monolayer on the imprinted polymer on the surface of MIP rather than involving multilayered adsorption sites. In addition, the result for *Q*_MAX_ values for MIP and NIP indicated that the loading capacity was relatively low. This is probably due to the low amount of acetonitrile employed during polymerization, as it was used as the porogen. That choice was made to prepare the materials in conditions as close as possible to the final application. In addition, the *Q*_MAX_ values were also different between MIP and NIP, with the one of NIP much lower than that of the MIP material. The difference could be attributed to the absence of imprinting sites for PAT on the NIP and was previously reported for other MIP materials [29, 38].

**Table 1:** Results of the thermodynamic and kinetic characterization of PAT rebinding to MIP and NIP materials. The parameters were obtained by fitting rebinding data to the Langmuir and Freundlich thermodynamic models and the pseudo-first-order or pseudo-second-order kinetic models. The constants and parameters are reported for each model with the corresponding coefficients of determination. Details on equations used for fitting are reported in the Supplementary Information

	MIP	NIP
Langmuir isotherm		
* Q*_Max_ (ng mg^−1^)	13.33	1.82
* K*_L_ (mL mg^−1^)	0.12	0.03
* R*^2^	0.98	0.96
Freundlich isotherm		
* K*_F_ (mL mg^−1^)	1.01	0.23
* n*	1.60	0.80
* R*^2^	0.56	0.92
Pseudo-first-order kinetics		
* K*_1_ (min^−1^)	0.04	9E04
* R*^2^	0.44	0.38
Pseudo-second-order kinetics		
* K*_2_ (min^−1^)	0.50	4.40
* R*^2^	1.00	1.00

Dynamic binding was investigated over a time range of 60 min. The saturation curve showed that complete adsorption occurred within 20 min (Fig S5B). The adsorption mechanism was further studied by linear fitting using pseudo-first-order and pseudo-second-order models (Fig S6, Table 1). The values of the coefficients of determination were calculated and used to assess the best fit, which was found for the second-order kinetic model for both MIP (*R*^2^ 0.996, Fig S6B) and NIP materials (Fig S7B).

### Selectivity investigation of MIP

The selectivity of the enrichment process was evaluated. 5-HMF is the most important interferent compound for PAT, especially when using UV detectors. As such, the selectivity for binding was investigated against this compound. The *K*_D_ values for PAT and the interferent were calculated as 8.8 and 1.9, respectively, showing a selectivity (*α*) of 4.62. The result indicated that the MIP material could bind PAT with higher selectivity than 5-HMF, which is beneficial in HPLC-DAD analysis to exclude the positive contribution from this molecule.

### Study of recovery from MIP material

The recovery percentage indicates the efficiency of the MIP in binding and releasing the target molecule, and it is an essential parameter for evaluating the performance of MIPs. The elution conditions were investigated to improve the recoveries. Initially, literature conditions were tested [42], and elution was performed with water/methanol/acetic acid (4:1:0.25, *v/v/v*); as PAT recovery was low (10 ± 2%), a second elution consisting of acetonitrile/acetic acid, 5:0.25 (*v/v*), was performed. The recovery was quantitative (93 ± 5%). In the final method, the first elution condition was used as a simple washing step, and only the second elution was analyzed to determine PAT. The elution with organic solvent and acid indicated that both reversed-phase and H-bonding interactions were involved in the interaction of PAT with the MIP material.

## Validation results

The validation of the analytical method for the analysis of PAT in apple fruit juices using the uric acid-imprinted MIP material was performed following the main FDA guidelines, using an apple juice pool. The validation results are reported in Table S2. The recovery (RE) values were calculated at three fortification levels (c_1_, 1 ppb; c_2_, 10 ppb; c3, 50 ppb, Table 2). These values were chosen based on the potential minimum contamination defined by the European Union. Allowable levels of PAT are 10 μg kg^−1^ in apple juice and solid apple-based products, including apple compote and puree, for infants and children. The high-level fortification at 50 ng mL^−1^ was chosen to meet the limit in spirit drinks, cider, and other fermented drinks derived from apples or containing apple juice (Commission Regulation (EC) No 1881/2006).

**Table 2:** Recovery (RE) and matrix effect (ME) results for PAT analysis in apple juice samples at three different concentrations. (c_1_, 1 ng mL^−1^; c_2_, 10 ng mL^−1^, c_3_, 50 ng mL^−1^). Results are displayed as mean ± standard deviation (*n* = 3)

Compound	RE% ± SD			ME% ± SD		
	c_1_	c_2_	c_3_	c_1_	c_2_	c_3_
Patulin	85 ± 6	90 ± 3	95 ± 1	98 ± 4	95 ± 2	87 ± 5

As a comparison, the recovery was evaluated for the NIP material as well, at the same concentration levels as described for the MIP material (Table S3). In this case, the recoveries were unsatisfactory (45–70%), especially at the high concentration level. The matrix effect was also larger than for the MIP material, probably due to unspecific interactions with matrix components.

The intraday and interday precision was evaluated by performing recovery experiments (*n* = 6) on the same day and for 6 consecutive days, measuring the Relative Standard Deviation (RSD). The RSD values were lower than 15%, as defined by the acceptance criteria of FDA guidelines (Table S2). The linear dynamic range was constructed by assessing the minimum allowable concentration and extending to higher values to evaluate the method’s linearity (1–100 ng mL^−1^). The coefficient of determination for linear regression (*R*^2^) was greater than 0.99.

The LOD was determined at 0.5 ng mL^−1^. The LOQ was set at the lower limit of the linear dynamic range (1 ng mL^−1^).

## Analysis of PAT in real samples

Results of the analysis of the 20 commercial apple fruit juices are reported in Table S4. The quantification is expressed in μg of PAT per mL of apple juice. All the analyzed samples showed PAT concentrations below the EU commission limit. In 17 samples, PAT was not detected (ND). Sample 5 exhibited a chromatographic peak corresponding to a non-quantifiable PAT amount (< LOQ). However, two samples tested positive for PAT, falling within the 1.89–1.02 ng mL^−1^ range (samples 9 and 14, respectively). The findings of this survey highlighted that some Italian apple juices are contaminated with PAT, as previously demonstrated by Piemontese et al. in 2005 [45]. The percentage of positive samples in their study was higher (48% compared to 15% in this study), indicating an improvement in consumer safety over the years. The chromatogram of sample 14 is reported in Supplementary Information Fig S8.

The method developed in this work was compared to previously reported methods using MIP enrichment or clean-up and HPLC-UV analysis for quantification of PAT in apple juice (Table 3). The polymer described in this work is new to materials previously developed and based on different organic polymers [29, 40, 46] obtained by radical polymerization with completely different monomers and inorganic polymers based on imprinting of silica [31, 38, 47]. These MIP materials were mostly prepared in high organic solvent conditions, with only one exception [47]. The adsorption capacity of the developed MIP material is lower than the one reported for previously developed MIP materials; therefore, larger amounts of sorbent were needed in our method for extraction. The extraction time was also generally longer than previously reported procedures based on SPE but faster than a similar procedure based on dispersed magnetic material [38]. As far as the validated methods were concerned, recoveries of the developed procedure were similar to previous methods. LOD and LOQ achieved in the present work were better compared to all developed MIP materials for PAT extraction, highlighting the effectiveness of our hydrophilic sorbent. Notably, the LOD value obtained in this work was similar to the SPE with a commercial material, although the latter was used for extraction not only of apple juice but also of apple puree and jam samples [36]. As a general final consideration, the developed method had a lower LOD than most works using HPLC-UV detection of PAT for which limits are at a concentration of 10 μg kg^−1^ or 10 μg L^−1^ [20].

**Table 3:** Comparison of the proposed method with the literature methods using MIP extraction or clean-up and HPLC-UV analysis for the determination of PAT in apple juice. For comparison, the table summarizes the type of material prepared in each work, the monomers and crosslinkers used for preparation of the MIP part of the final material, the template used for imprinting, the solvent in which the MIP was prepared, the type of analytical procedure used for extraction of PAT, the adsorption capacity of the MIP, and the extraction time associated with the MIP procedure and validation results for LOD, LOQ, and recovery of the final methods. Template, MIP composition, and additional information were not available for commercial materials

Material type	Monomers (cross-linker)	Template	Solvent	Extraction technique (sorbent amount)	*Q*_MAX_	Extraction time	LOD	LOQ	RE (%)	Ref
SiO_2_@MIP	Maleic acid (EGDMA^1^)	Patulin	Acetonitrile	SPE (50 mg)	261.8 μg g^−1^	10 min	8.6 μg L^−1^	28.6 μg L^−1^	82–98	[29]
EASIMIP™	Commercial material	-	-	SPE	-	-	6 μg L^−1^	20 μg L^−1^	81–110	[30]
Magnetic-SiO_2_	APTES^2^ (TEOS^3^)	2-oxindole	87% methanol	MSPE^4^ (20 mg)	1.97 mg g^−1^	105 min	3 μg kg^−1^	10 μg kg^−1^	86–96	[38]
SiO_2_	TEOS	Isatin	Water	SPE online 1g	-	7 min	11 ng mL^−1^	-	> 88	[47]
AFFINISEP	Commercial material	-	-	SPE	-	-	0.6 μg kg^−1^	-	73–108	[36]
SiO_2_	APTES (TEOS)	Oxindole	83% methanol	SPE online (50 mg)	1.657 mg g^−1^	27 min	0.5 μg L^−1^	2 μg L^−1^	60–98	[31]
SiO_2_@MIP	Acrylamide (EGDMA)	6-hydroxynicotinic acid	Acetonitrile	SPE (180 mg)	0.654 μg mg^−1^	20 min	10 μg L^−1^	40 μg L^−1^	90–96	[46]
MIP	Methacrylic acid (EGDMA)	Oxindole	Ethyl acetate	SPE (400 mg)	5.7 mg g^−1^	24 min	5 μg g^−1^	6 μg g^−1^	84–89	[40]
MIP	Melamine, dopamine (formaldehyde)	Uric acid	8% acetonitrile	d-SPE (100 mg)	13.33 ng mg^−1^	40 min	0.5 ng mL^−1^	1.0 ng mL^−1^	85–90	This work

*EGDMA* ethylene glycol dimethacrylate, *APTES* (3-aminopropyl) triethoxysilane, *TEOS* tetraethyl orthosilicate, *MSPE* magnetic SPE

Finally, the comparison was extended to other procedures, including SPE on commercially available polyvinylpolypyrrolidone-florisil sorbent, μ-QuEChERS, and LLE procedures. Câmara and his co-workers [19] developed a μ-QuEChERS/LC-MS/MS method for PAT extraction, for which limits were relatively lower than the ones obtained in our work (0.32 vs. 0.5 ng mL^−1^). Despite the lower LOD and LOQ, QuEChERS methodology requires subsequent clean-up of the obtained extract that extends preparation time and cost of analysis. Instead, an LLE extraction using acetonitrile offered acceptable results suggesting that it is adequate for quantifying PAT in apple juice but with LOD and LOQ higher than our proposed methodology (3.5 vs. 0.5 ng mL^−1^) [15]. The same considerations apply to the SPE pretreatment method using a home-made polyvinylpolypyrrolidone-florisil column [23]. Although the developed method can be competitive with the above-cited methods, the use of HPLC-UV detection is less effective than other strategies, previously described, and particularly sensitivity can be significantly improved, down to pg mL^−1^ level by using sensors and especially MIP-based sensors [48].

### Confirmation with UHPLC-MS/MS

**Fig. 5:**
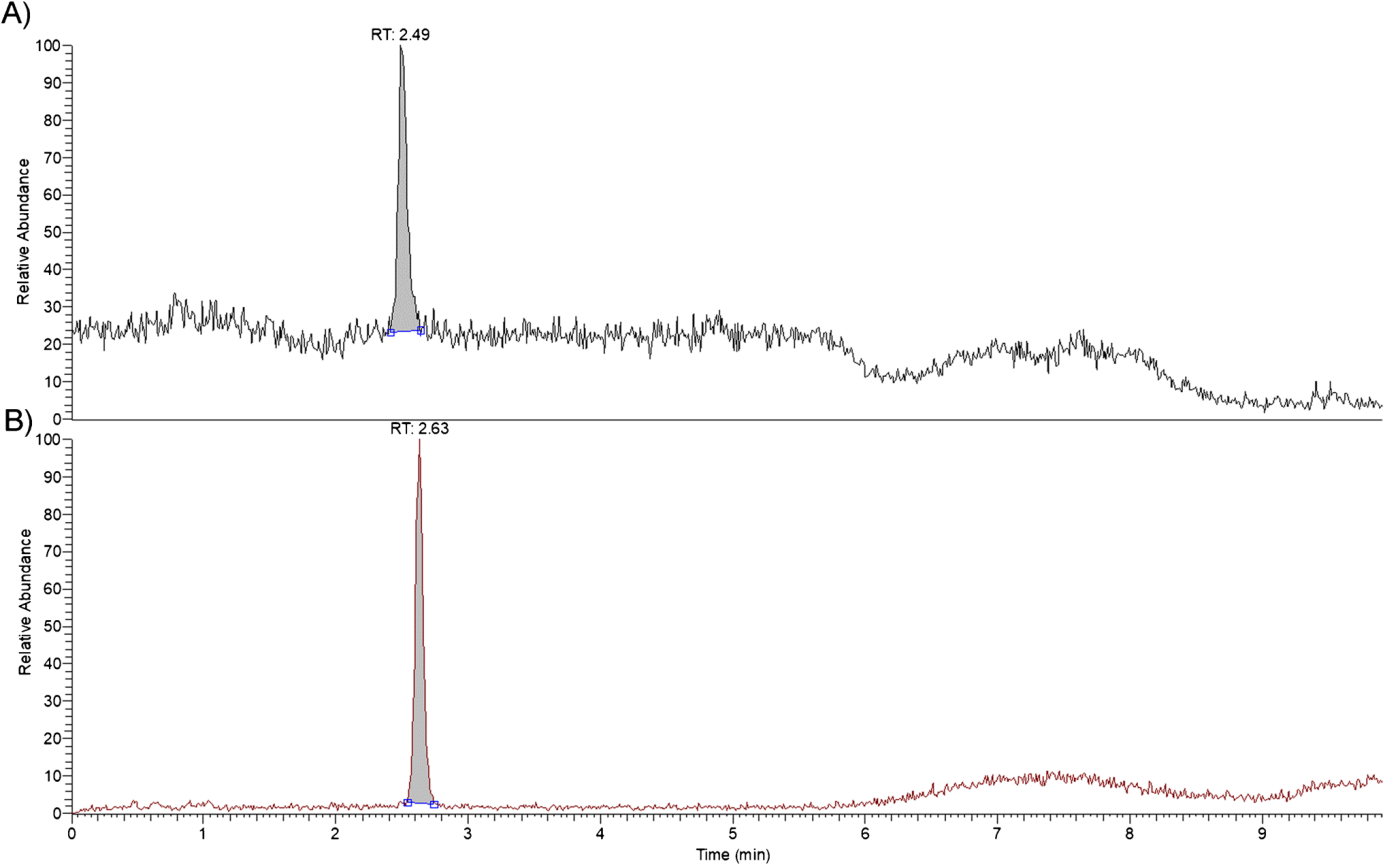
UHPLC-MS/MS chromatogram for 5-HMF (**A**) and PAT (**B**). Total ion chromatogram (TIC) of the transitions for 5-HMF (**A**) and PAT (**B**). Example of apple juice sample fortified with PAT and 5-HMF after extraction at 5 ng mL^−1^

HPLC-UV detection is the official method for determining PAT, as described by the Association of Official Analytical Chemistry, item 995.10 [49]. With this method, 5-HMF interference can occur in determining PAT in apple juices and derived products. Moreover, 5-HMF is present at levels two or three times higher than PAT, which can cause a severe problem in determining PAT in biological samples [50]. The identity of PAT in the two positive samples was confirmed by UHPLC-MS/MS, as described in the Supplementary Information. In particular, the chromatographic methods were developed to ensure a baseline separation between PAT and 5-HMF (Fig. 5).

PAT was identified by comparison with certified reference standards, and two transitions were monitored: one quantifier (most intense) and one qualifier (less intense, Table S1). The agreement between standard compounds and native compounds was assessed by matching the retention time, selected transitions, and ion ratio between the transitions. The presence of PAT in apple juice samples was confirmed (a representative chromatogram of PAT positive sample is reported in Fig S9). For 5-HMF, its protonated precursor ion [M+H]^+^ and two of its transitions [127→81 (qualifier); 127→109 (quantifier)] were monitored, with the ion ratio between the transitions being 38. On the other hand, for PAT, the ion ratio was 25.

## Conclusions

In the present study, for the first time, a novel hydrophilic MIP based on dopamine-formaldehyde–melamine was prepared in a water/acetonitrile, 92:8 (*v*/*v*) mixture for PAT extraction. Dopamine and melamine were used as double-functional monomers and formaldehyde as a cross-linker, introducing an abundance of hydrophilic groups. Moreover, uric acid was chosen as a dummy template for its structural similarity to PAT and its non-toxic nature compared to patulin as the template used in previously developed materials for PAT enrichment. The material was extensively characterized and studied to elucidate the thermodynamics and kinetics of the binding process. Langmuir’s model better described the adsorption of PAT on the material. The kinetic study indicated a rapid absorption occurring within 20 min. The analytical methodology was validated and showed good recovery, LOD, and LOQ values; the method was found suitable for the analysis of PAT in apple juices. In addition, the material had sufficient selectivity for PAT against the most common interference in apple juices, i.e., 5-HMF. From the analysis of apple juice commercial samples, two samples were found contaminated with PAT. It may be concluded that the method fulfilled all of the validation criteria, i.e., it is appropriate for routine surveillance of PAT in apple juice. From a perspective, it is reasonable to apply the new material to similar apple-based products, such as fruit juices. The method is competitive with other previously described methods, although improvements could include a detailed study to increase the surface area of the material and application with other more sensitive detection systems.

### Supplementary information

ESM 1: Figures S1-S9 and Tables S1-S4 (DOCX 13274 kb)
