# Supplementary material for: Dispersive solid phase extraction using a hydrophilic molecularly imprinted polymer for the selective extraction of patulin in apple juice samples
Source: Mikrochim Acta. 2023 Nov 25;190(12):485. doi: 10.1007/s00604-023-06056-8 (PMC10676307; doi:10.1007/s00604-023-06056-8)
Supplement: Supplementary file 1 — Figures S1-S9 and Tables S1-S4 (DOCX 13274 kb) [file 604_2023_6056_MOESM1_ESM.docx]

**Supplementary Information**

**Dispersive-solid‐phase extraction using a hydrophilic molecularly imprinted polymer for the selective extraction of patulin in apple juice samples**

Chiara Cavaliere, Andrea Cerrato, Aldo Laganà, Carmela Maria Montone*, Susy Piovesana Enrico Taglioni, Anna Laura Capriotti

Dipartimento di Chimica, Università di Roma La Sapienza, Piazzale Aldo Moro 5, 00185 Roma, Italy

***Corresponding author**: Carmela Maria Montone

Dipartimento di Chimica, Università degli Studi di Roma La Sapienza

Piazzale Aldo Moro 5

00185 Rome, Italy

E-mail: [carmelamaria.montone@uniroma1.it](mailto:carmelamaria.montone@uniroma1.it)

T (+39) 06 4991 3945

**Adsorption experiments**

**Adsorption isotherm**

The adsorption performance of MIP for PAT was studied as follows. A fixed amount (2 mg) of MIP and NIP was dispersed in 1.5 mL of aqueous solution with different concentrations of PAT (5–350 ng mL^-1^). After that, samples were sonicated for 30 min at room temperature to reach equilibrium. The equilibrium amounts of PAT adsorbed on the material, Q_e_ (mg g^-1^), were calculated with the following equation, Eq. (1):

$Qe=\frac{\left( C_{0}-Ce \right)V}{m}$ Eq. (1)

where C_0_ (mg mL^-1^) indicates the initial concentration of PAT, while Ce (mg mL^-1^) is the equilibrium concentration; V (mL) and m (g) are the volumes of PAT solution, and the mass of MIP or NIP materials. The pH of the solution was acidic (0.1% acetic acid) for all the experiments. The adsorption process was evaluated by applying the Langmuir and Freundlich isotherm models to the experimental data. For the Langmuir fit, Eq. (2) was used:

$Qe=\frac{Q_{MAX}K_{L}C_{e}}{1+K_{L}C_{e}}$ Eq. (2)

Qe was the amount of PAT adsorbed per unit of adsorbent, Ce was the equilibrium supernatant concentration, K_L_ was the adsorption-free energy constant, and Q_MAX_ was the maximum adsorption capacity. Fitting Ce/Qe vs. Ce, Q_MAX_, and K_L_ can be extrapolated. For the Freundlich fit, the equation below was used, Eq. (3):

$Qe=K_{F}{C_{e}}^{\frac{1}{n}}$ Eq. (3)

where K_F_ was the Freundlich constant indicative of the relative adsorption capacity of the adsorbent, and 1/n was the heterogeneity factor. Linear fitting of logQ_e_ vs logC_e_ allows the determination of constants n and K_F_. All experiments were performed in duplicate analysis, and the concentration of PAT was measured by HPLC-DAD.

**Dynamic adsorption**

The dynamic adsorption experiments were carried out as previously described for the static adsorption isotherm experiments [1]. A fixed concentration of PAT at the saturation level (20 ng mL^-1^ for the MIP material, 10 ng mL^-1^ for the NIP material) was used, incubated at room temperature with the two materials (10 mg for MIP, 2 mg for NIP), and the supernatants were analyzed at different time points (0-60 min). Eq. (4) allowed us to calculate the amount of PAT at each time point:

$Qt=\frac{\left( C_{0}-Ct \right)V}{m}$ Eq. (4)

where Q_t_ was the amount of PAT adsorbed per unit of adsorbent at each time point, and C_t_ represents the supernatant concentration at each time point.

The kinetic of adsorption was investigated by application of pseudo-first-order and pseudo-second-order kinetic models. According to Eq. (5) and Eq. (6), respectively:

$log\left( Qe-Qt \right)=logQe-(\frac{K_{1}}{2.303})t$ Eq. (5)

$\frac{t}{Q_{t}}=\frac{1}{K_{2}Q_{e}^{2}}+\frac{t}{Q_{e}}$ Eq. (6)

where t is the time point (seconds), K_1_ is the pseudo-first-order adsorption constant, and K_2_ is the pseudo-second-order adsorption constant. All experiments were performed in duplicate analysis, and the concentration of PAT was measured by HPLC-DAD.

**Selectivity evaluation**

The experiments were performed by loading 1 mL of 25 ng mL^-1^ standard solution of PAT and 5-HMF on 100 mg of MIP material for 30 minutes at room temperature. After incubation, the supernatant was analyzed to determine the residual amount of PAT and 5-HMF. HPLC-MS analysis was performed as described in the Supplementary Information (High-performance liquid chromatography-tandem mass spectrometry). The distribution ratios (K_D_) and selectivity coefficients (α) were calculated as described by Eq. (7) and Eq. (8):

$K_{D}=\frac{V*(C_{0}-C_{e})}{C_{e}*m}$ Eq. (7)

$\alpha=\frac{K_{D}^{PAT}}{K_{D}^{5-HMF}}$ Eq. (8)

V is the loaded volume, C_0_ the original concentration, C_e_ the measured concentration of the supernatant after 30 minutes, m the amount of MIP.

**High-performance liquid chromatography-tandem mass spectrometry**

UHPLC-MS/MS analysis was performed on an Ultimate 3000 binary pump (Thermo Fisher Scientific in Bremen, Germany) and a triple quadrupole mass spectrometer (TSQ Vantage EMR, Thermo Fisher Scientific, Bremen, Germany) connected via a heated electrospray (HESI) source. Xcalibur^TM^ v.2.2 software (Thermo Fisher Scientific, Bremen, Germany) was employed to manage, acquire, and process LC-MS data. The separation was performed on a Hypersil Gold Vanquish column (2.1 × 100 mm, 1.9 µm particle size, Thermo Scientific) equipped with a Hypersil Gold C18 pre-column (2.1 × 4 mm, 5 µm particle size, Thermo Scientific). The column temperature was maintained at 40 °C, and the flow rate was set at 0.4 mL min^-1^. The mobile phases were water (A) and acetonitrile (B), both with 0.1% (*v*/*v*) acetic acid. The gradient profile was as follows (t in minutes): t_0_, B = 2%; t_4_, B = 2%; t_7_, B = 80%; t_8_, B = 80%; t_8.5_, B = 2%, t_10_, B = 2%. The HESI source was set at -2.6 kV and 3.0 kV spray voltages for negative and positive ionization modes, respectively. The vaporizer temperature was maintained at 290 °C, while the capillary (ion transfer tube) temperature was set to 280 °C. The sheath gas pressure, ion sweep gas pressure, and auxiliary gas pressure were configured at 40, 0, and 20 (arbitrary units), respectively. Mass calibrations and resolution adjustments were automatically carried out on quadrupoles and resolution lenses using the manufacturer's solution once a month to ensure accuracy. For each compound, a solution at a concentration of 1 µg mL^-1^ was infused at a flow rate of 10 µL min^-1^, and at least two selected reaction monitoring (SRM) transitions were monitored to optimize the parameters for analyzing the analytes (Table S1).

**Table S1**: Chemical formulas, precursor and product ions, and MS parameters of PAT, 5-HMF, and caffeine (trimethyl-^13^C_3_) standards. Quantifier transitions are marked in bold.

| **Compound** | **Chemical formula** | **Precursor ion** | **Product ions m/z** | **Collision Energy**  **CE** | **S-lens voltage (Hz)** | **Ionic ratio average^1^** |
| --- | --- | --- | --- | --- | --- | --- |
| Patulin | C_7_H_6_O_4_ | [M-H]^-^153 | 81  **109** | 15  13 | 50 | 25 |
| 5-HMF | C_6_H_6_O_3_ | [M+H]^+^  127 | 81  **109** | 16  7 | 46 | 38 |
| Caffeine-(trimethyl-^13^C_3_) | ^13^C_3_C_5_H_10_N_4_O_2_ | [M+H]^+^  198 | **140**  112 | 18  23 | 79 | 20 |

^1^The relative intensities between the qualifier and the quantifier transitions are reported as percentages.

**Method validation**

The HPLC-DAD method was validated following FDA guidelines, using an apple fruit juice pool. The parameters evaluated were: recovery (RE), matrix effect (ME), precision, linear dynamic range, linearity, and the limits of detection (LOD) and quantification (LOQ).

Recovery was calculated at three different concentrations (c_1_: 1 ng mL^-1^; c_2_ 10 ng mL^-1^; c_1_: 50 ng mL^-1^), and sample aliquots were extracted as described in paragraph *Molecularly imprinted dispersive solid-phase extraction of apple juice samples*. The first aliquot was fortified before extraction (C_1_), and the second aliquot after extraction (SIM). The recovery was calculated by comparing the areas (Eq. 9). The area of the PAT standard was always normalized to the area of the volumetric standard phenylalanine-d8 (Area VS).

$RE = \frac{\frac{Area C_{1}}{Area VS}-\frac{{Area C}_{0}}{Area VS}}{\frac{Area SIM}{Area VS} -\frac{{Area C}_{0}}{Area VS}} x 100$ Eq. (9)

C_0_ corresponds to the endogenous amount of PAT present in the samples; in our case, the endogenous amount was below the LOD.

The matrix effect was calculated by comparing the areas of SIM with a reference sample consisting of analytes alone in solvent without the matrix (RIF) (Eq. 10).

$ME = \frac{\frac{Area SIM}{Area VS}-\frac{{Area C}_{0}}{Area VS}}{\frac{Area RIF}{Area VS}} x 100$ Eq. (10)

Calibration curves were prepared in solvent by adding a known amount of analyte in the range of 1-100 ng mL^-1^. The intraday and interday precision was evaluated by performing recovery experiments (n = 6) at the c_2_ spiking level on the same day and for six consecutive days, measuring the Relative Standard Deviation (RSD).

LOD was calculated using the signal-to-noise ratio (S/N) equal to 3 method and verified to test the instrumental response. The LOQ was set at the lowest limit of the linear dynamic range.

All samples were analyzed in triplicate, and the results were averaged.


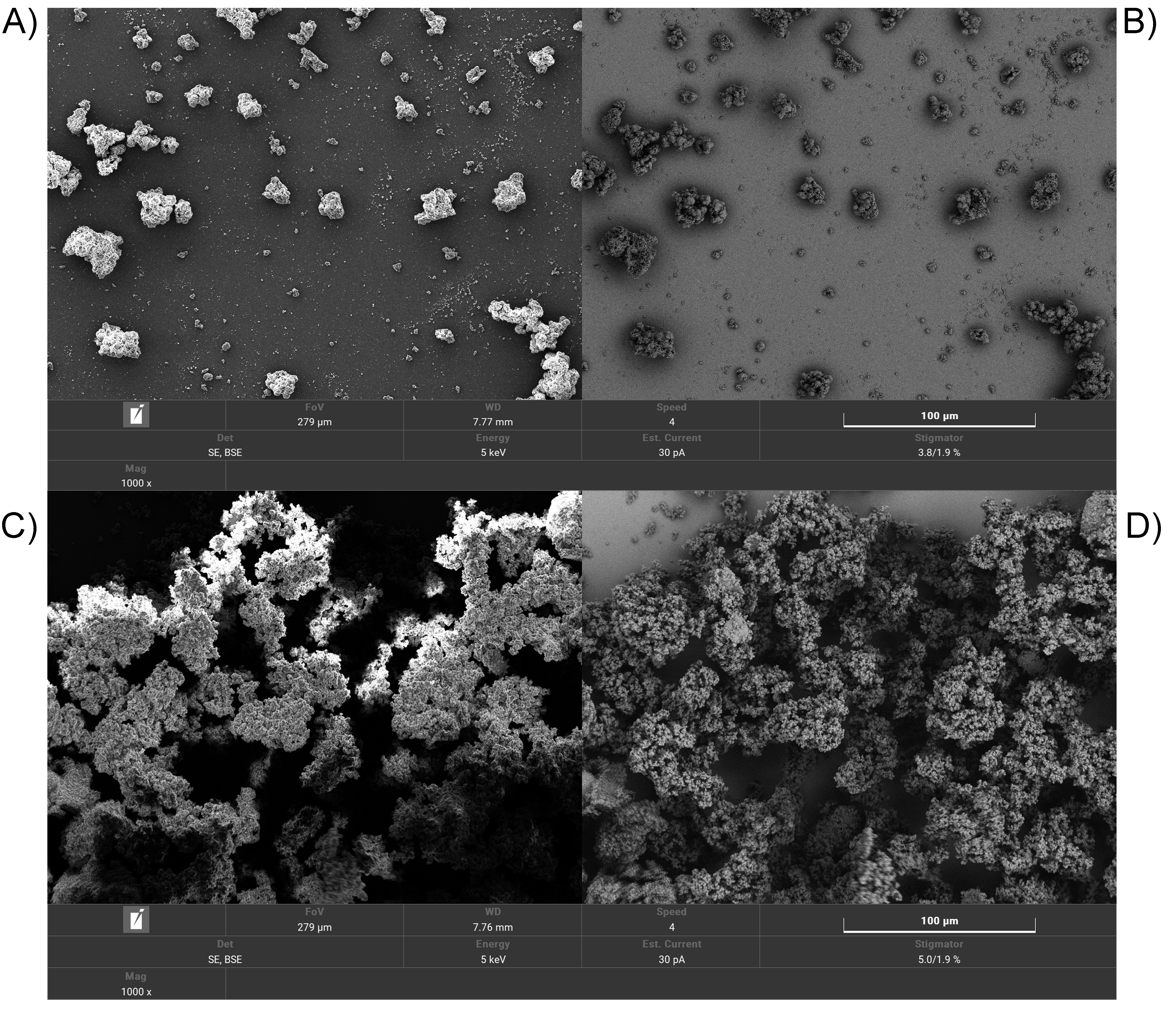


**Fig S1.** SEM images of the MIP (A, B) and NIP (C, D) materials at 1000 magnification. A) and C) show pictures obtained by using a secondary electron detector; B) and D) show the same images using a backscattered electron detector.

**
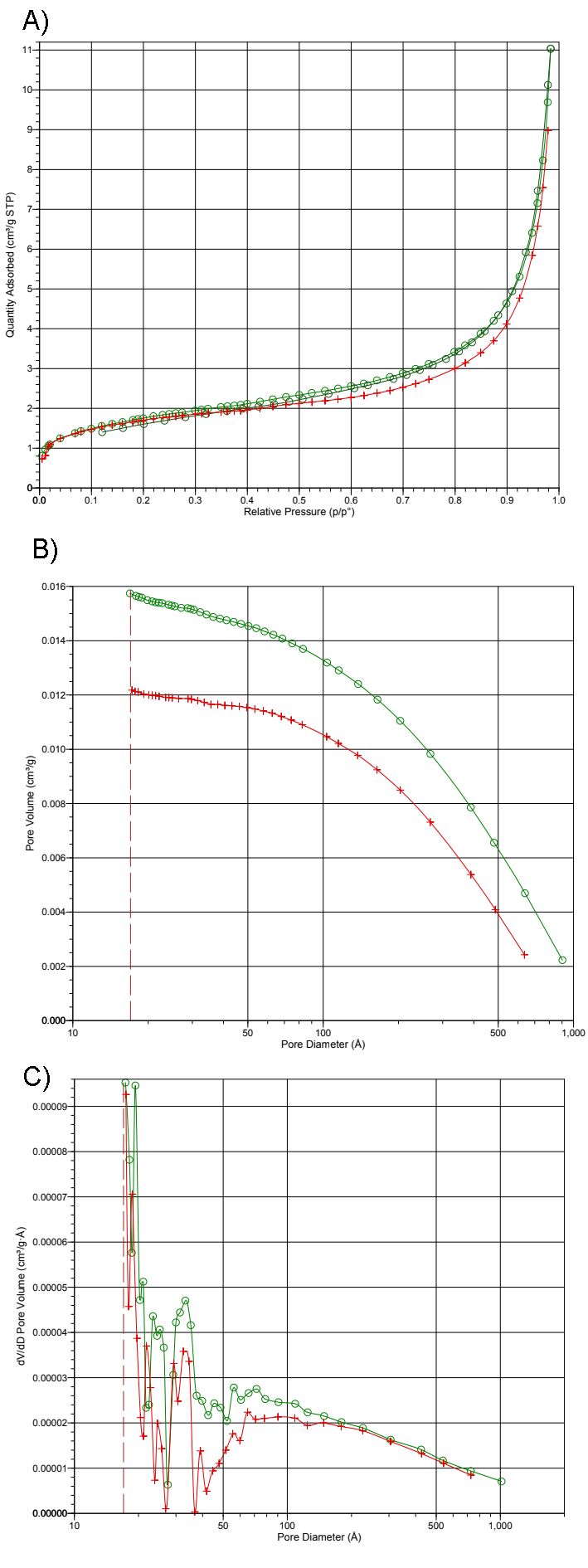

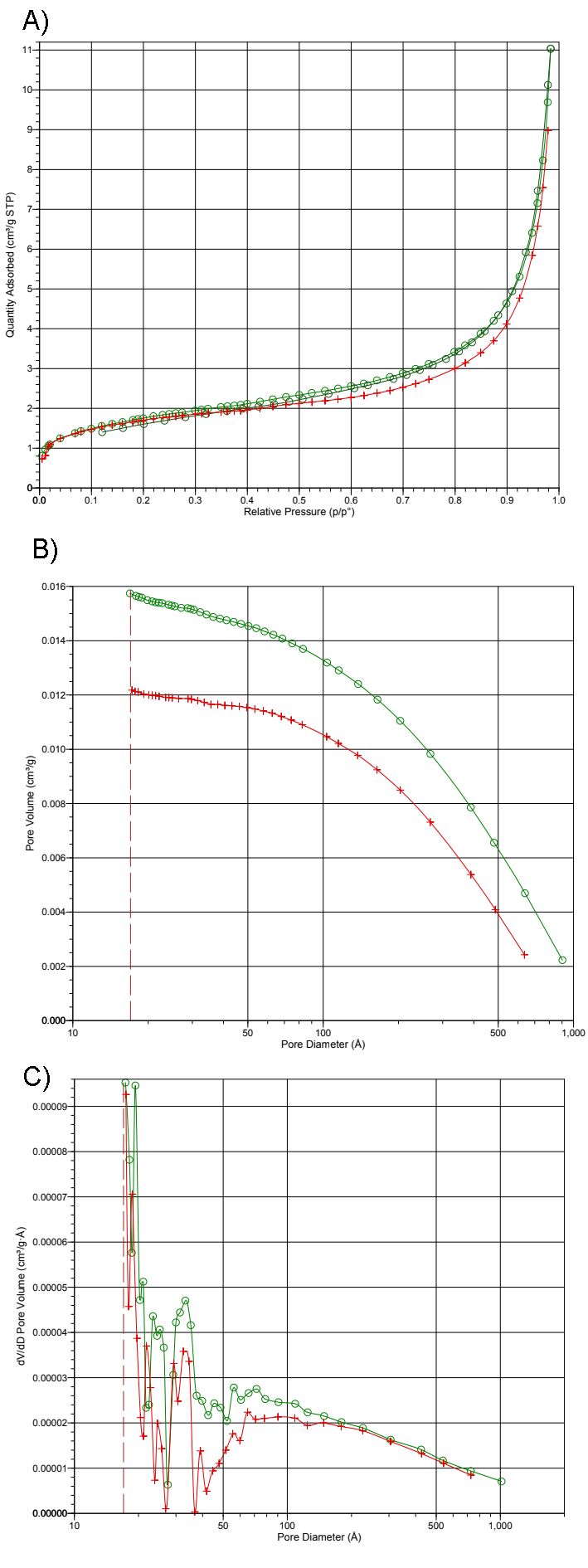
**


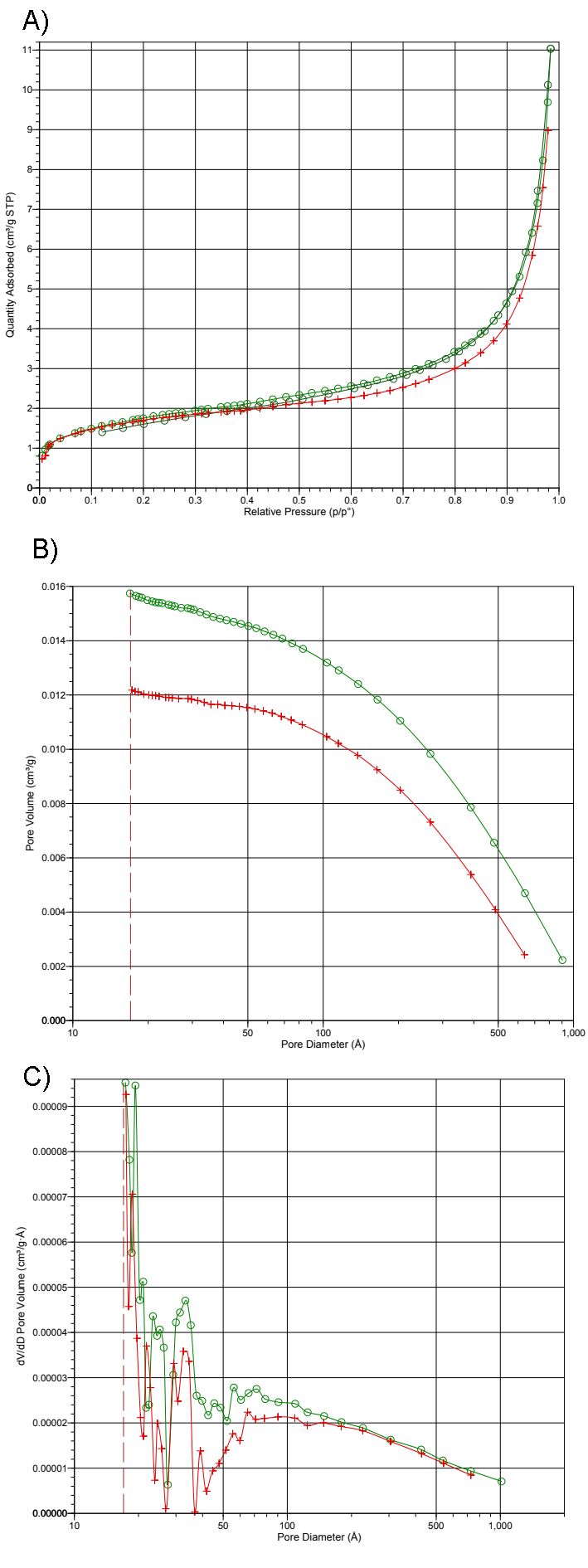


**Fig S2.** Results of BET analysis showing the A) isotherm linear plots, B) the BJH adsorption cumulative pore volume (larger), and C) BJH adsorption dV/dD pore volume graphs. Red lines display the data for NIP material; green lines display results for MIP material.


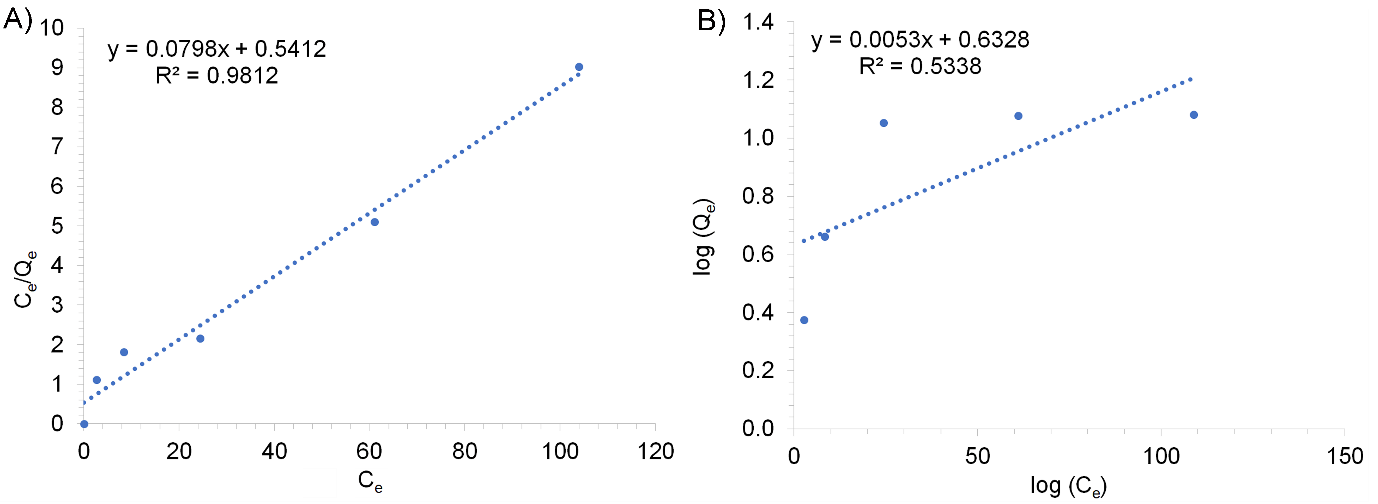


**Fig S3**. Static rebinding of PAT to the MIP material. Graphs are displayed for the application of the Langmuir (A) and Freundlich (B) models to the rebinding experimental data as described in the Supplementary Information. Equation results of the linear fittings, with the related coefficients of determination, are reported in the top part of each figure.


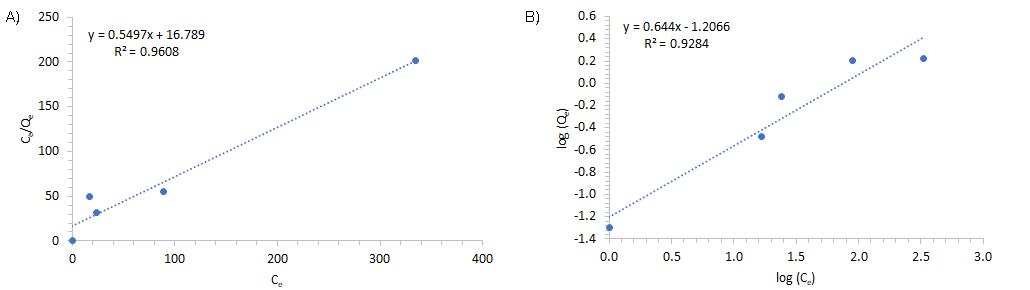


**Fig S4**. Static rebinding of PAT to the NIP material. Graphs are displayed for the application of the Langmuir (A) and Freundlich (B) models to the rebinding experimental data as described in the Supplementary Information. Equation results of the linear fittings, with the related coefficients of determination, are reported in the top part of each figure.


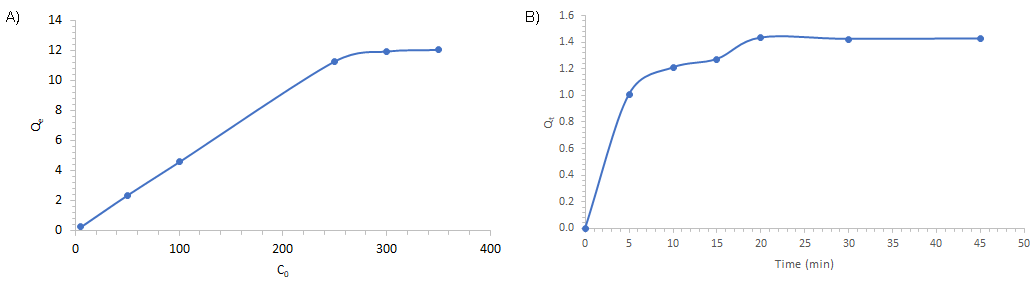


**Fig S5.** A) Thermodynamic adsorption trend as a function of the loaded amount of PAT on the MIP material; B) kinetic adsorption trend of PAT as a function of time on the MIP material.


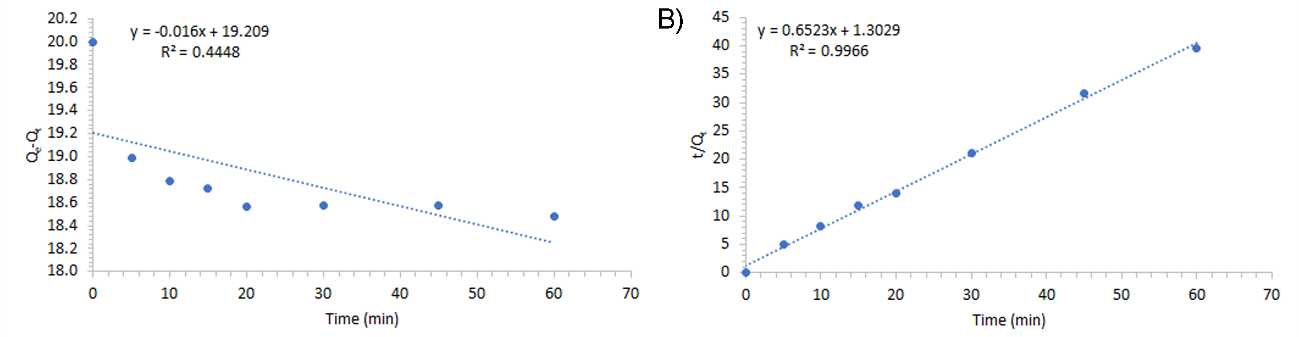


**Fig S6**. Results for the fitting of experimental data using a pseudo-first-order (A) or pseudo-second-order (B) kinetic model for the rebinding of PAT to the MIP material. Equation results of the linear fittings, with the related coefficients of determination, are reported in the top part of each figure.


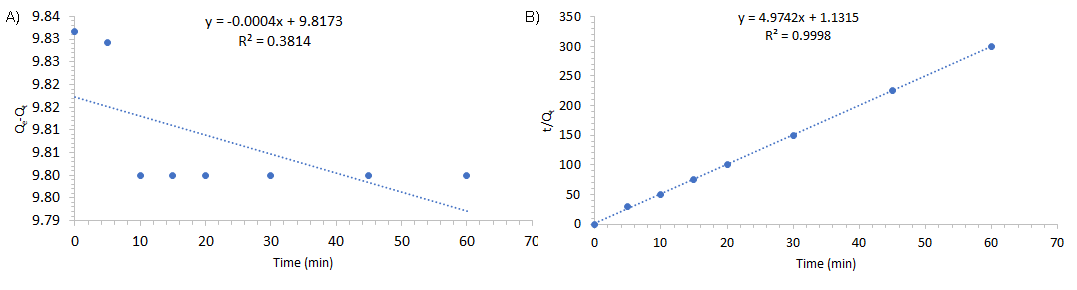


**Fig S7.** Results for the fitting of experimental data using a pseudo-first-order (A) or pseudo-second-order (B) kinetic model for the rebinding of PAT to the NIP material. Equation results of the linear fittings, with the related coefficients of determination, are reported in the top part of each figure.

**Table S2**. Analytical validation parameters for the developed method for analysis of PAT in apple juices using the uric-acid-imprinted MIP. RSD% for intraday and interday precision was evaluated by performing recovery experiments (n = 6) on the same day and for six consecutive days, at 10 ng mL^-1^.

| Compound | RSD% (Intraday) | RSD% (Interday) | linear dynamic range (ng mL^-1^) | R^2^ | LOD  (ng mL^-1^) | LOQ  (ng mL^-1^) |
| --- | --- | --- | --- | --- | --- | --- |
| Patulin | 10 | 11 | 1-100 | 0.9999 | 0.5 | 1 |

**Table S3**. Recovery (RE) and matrix effect (ME) for the analysis of PAT in apple juice samples at three different concentrations. (c_1_: 1 ng mL^-1^; c_2_ 10 ng mL^-1^, c_3_ 50 ng mL^-1^) for the NIP material. Results are displayed as mean ± standard deviation (n = 3).

| Compound | RE% ± SD | | | EM% ± SD | | |
| --- | --- | --- | --- | --- | --- | --- |
|  | c_1_ | c_2_ | c_3_ | c_1_ | c_2_ | c_3_ |
| Patulin | 70 ± 6 | 64 ± 5 | 45 ± 4 | 80 ± 2 | 76 ± 8 | 78 ± 4 |

**Table S4.** Results for the quantification of PAT in 20 commercial apple juice samples using the validated method based on uric-acid-imprinted MIP. The quantification is expressed in µg of PAT per mL of apple juice. Results are provided as mean value ± standard deviation (n = 3).

| **Sample** | **PAT (ng mL^-1^) Mean ± SD** |
| --- | --- |
| Sample 1 | -^a^ |
| Sample 2 | -^a^ |
| Sample 3 | -^a^ |
| Sample 4 | -^a^ |
| Sample 5 | <LOQ^b^ |
| Sample 6 | -^a^ |
| Sample 7 | -^a^ |
| Sample 8 | -^a^ |
| Sample 9 | 1.89 ± 0.28 |
| Sample 10 | -^a^ |
| Sample 11 | -^a^ |
| Sample 12 | -^a^ |
| Sample 13 | -^a^ |
| Sample 14 | 1.02 ± 0.12 |
| Sample 15 | -^a^ |
| Sample 16 | -^a^ |
| Sample 17 | -^a^ |
| Sample 18 | -^a^ |
| Sample 19 | -^a^ |
| Sample 20 | -^a^ |

^a^ PAT concentration was below LOD (0.5 ng mg^-1^);

^b^ PAT concentration was below LOQ (1 ng mL^-1^)


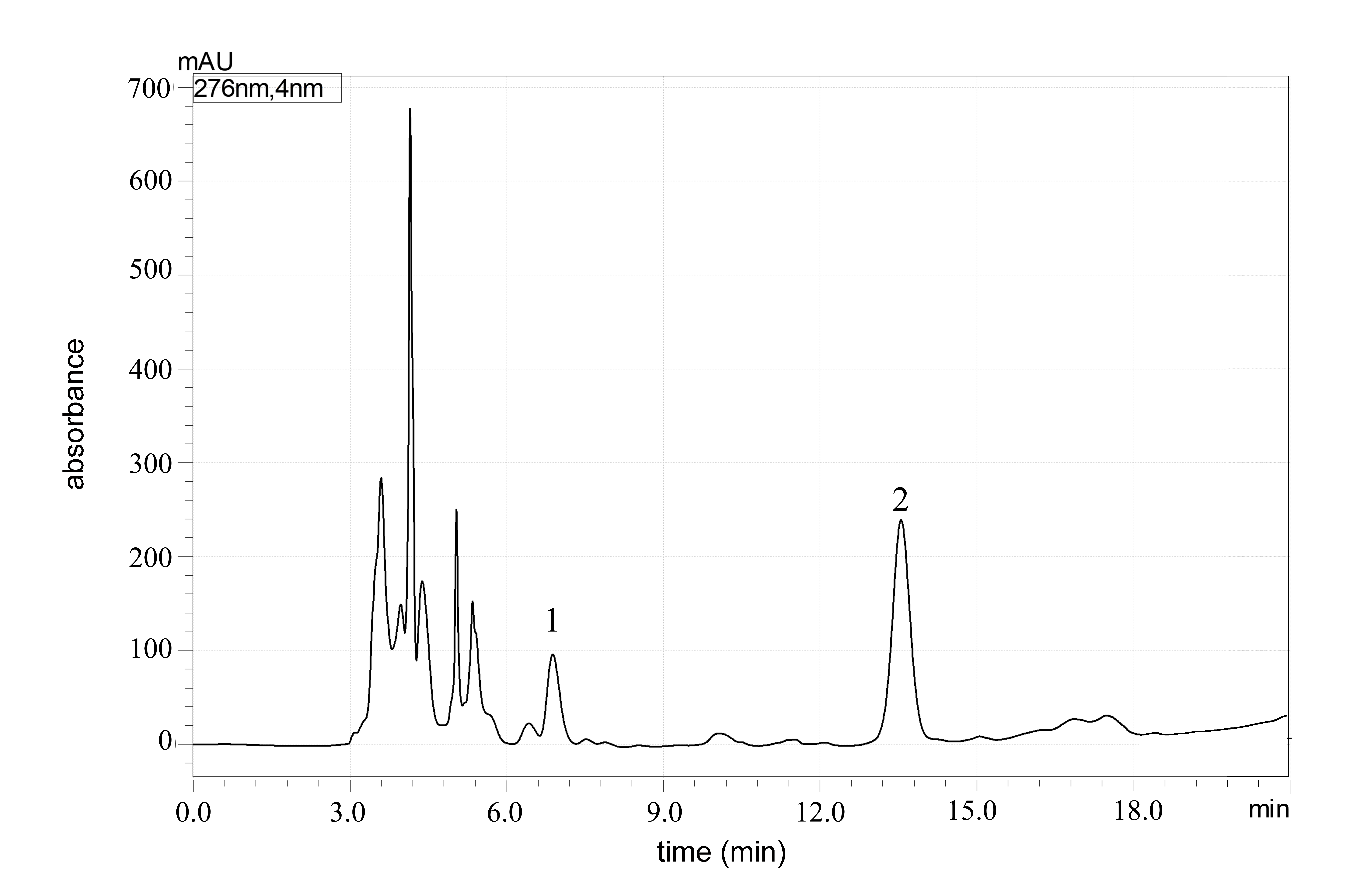


**Fig S8.** HPLC-DAD chromatogram for apple juice sample 14 with marked peaks of phenylalanine-d8 volumetric standard (1) and PAT (2).


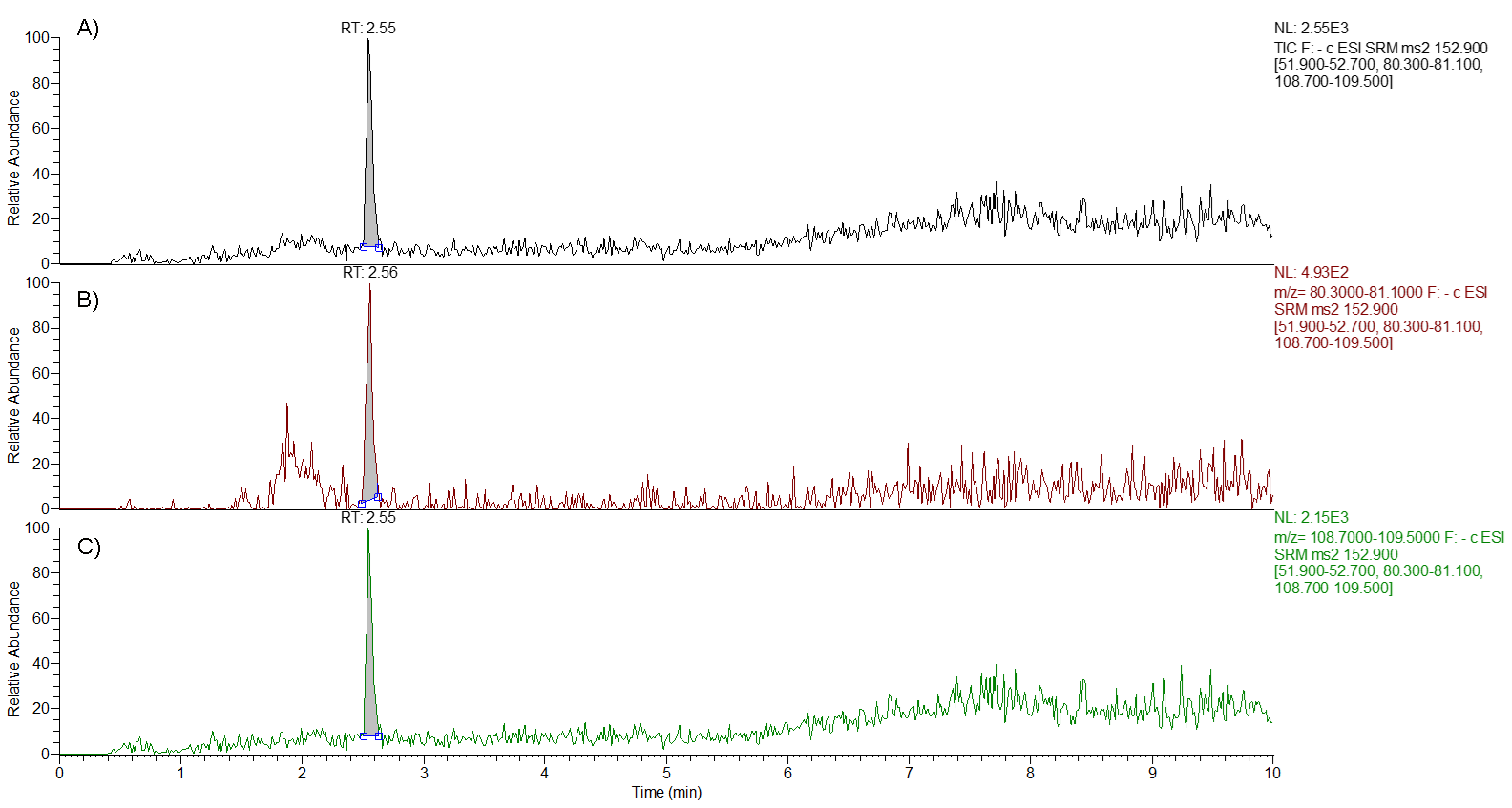


**Fig S9.** UHPLC-MS/MS chromatogram of apple fruit sample positive for PAT. A) Total Ion Chromatogram (TIC) of the MRM transitions of PAT; B) Extracted ion chromatogram of the *Qualifier* transition of PAT (81 *m*/*z*); C) Extracted ion chromatogram of the *Quantifier* transition of PAT (109 *m*/*z*).

**Refrences**

[1] C.M. Montone, A.L. Capriotti, C. Cavaliere, A. Cerrato, B. Giannelli Moneta, E. Taglioni, A. Laganà, A green extraction method based on carbon nitride sorbent for the simultaneous determination of free and conjugated estrogens in milk, Green Anal. Chem. 5 (2023) 100055. doi:10.1016/j.greeac.2023.100055.
